# Learning adaptability facilitates self-regulated learning at school: the chain mediating roles of academic motivation and self-management

**DOI:** 10.3389/fpsyg.2023.1162072

**Published:** 2023-05-25

**Authors:** Chunmei She, Qiao Liang, Wenjun Jiang, Qiang Xing

**Affiliations:** School of Education, Teachers College, Guangzhou University, Guangzhou, China

**Keywords:** learning adaptability, self-management, self-regulated learning, “double reduction”, academic motivation

## Abstract

Studies have demonstrated that learning adaptability has emerged as an important factor for students’ utilization of self-regulated learning for successful learning, but how this association occurred is not clear yet. This study aimed to clarify the mechanism of the relationship between learning adaptability and self-regulated learning by investigating the chain mediating roles of academic motivation and self-management of 787 junior high school students under the “double reduction” background. The results showed that (1) learning adaptability had significant positive effects on junior high school students’ self-regulated learning and (2) academic motivation and self-management played independent and accumulative mediating roles in the relationship between learning adaptability and self-regulated learning. These findings help to understand how to support students in successfully coping with the new challenges brought by educational reform and promote effective adjustment to challenges, including the “double reduction.” The key contribution of this study is to provide new insights into the literature that academic motivation and self-management separately and sequentially mediate the learning adaptability, self-regulated learning links, and learning adaptability is effective driver of self-regulated learning in the population of junior high school students.

## 1. Introduction

With the implementation of the “double reduction” policy, China’s primary and secondary education is facing new reforms. For junior high school students, the new policy means that they will be freed from heavy homework and the burden of time-consuming off-campus training; and have more spare time, but at the same time it also poses new challenges to students’ learning adaptability and autonomous learning ability. Junior high school students will face the new problems of adapting to rapid changes in the learning environment as soon as possible and taking the initiative to adopt appropriate learning strategies for learning. Learning adaptability, based on cognitive-developmental theory, as a form of self-regulation in challenging contexts (Collie and Martin, 2017), refers to the ability of learners to actively adjust their learning strategies and behaviors based on the learning environment provided by the school (curriculum, teachers, teaching equipment, teaching support, etc.) on the premise of an existing knowledge base and knowledge structure to coordinate their learning psychology and behavior with the changing learning environment to achieve good learning achievements (Nan, 2021).

Learning adaptability has already been shown to have a great theoretical (Zhang, 2017) and practical (Raza et al., 2021; Wang et al., 2021) influence on students’ learning results, subsequent educational choices, and opportunities as well as their mental well-being (Luo et al., 2018; Zhang and Peng, 2020). Ascertaining its role in the learning process may provide key implications for interventions to facilitate successful learning in novel or uncertain academic environment, given the influence of learning adaptability on students’ academic development. Many studies paid attention to the effect of (learning) adaptability on students’ self-regulated learning (SRL) (Xu et al., 2021; Feraco et al., 2022; Guo, 2022). Self-regulated learning, defined as a deliberate cyclical process of three phases (a preparatory, a performance and a reappraisal phase), in which learners proactively and independently apply multiple strategies to set realistic and specific learning goals, maintain their motivation, regulate their emotions, and monitor and evaluate their progress toward the learning goals (Zimmerman and Moylan, 2009), the largest variance was explained by learning adaptability compared to online self-efficacy and sources of stress (Stan et al., 2022). Despite the evidence for the important role of learning adaptability played in students’ SRL, several research gaps deserve further exploration, especially in relation to different aspects of learners’ learning. First, concerning associations between learning adaptability and SRL in the Chinese “double reduction” context, previous studies mainly focused on college students in the context of online learning (Otaki et al., 2022; Stan et al., 2022). However, little of the above associations are known between learning adaptability and SRL among junior high school students whose academic adaptation is still unsatisfactory in the face of the teaching reform brought about by the new deal (Liu, 2021). Weak learning adaptability affects the capacity to activate regulating strategies (Stan et al., 2022) and low SRL activity achieved significantly worse learning outcomes than students with higher SRL activity (van Alten et al., 2021). Therefore, it is vital to investigate the role of learning adaptability in the development of SRL among junior high school students. Second, the intervening mechanisms underlying the link between learning adaptability and SRL are not clear yet. The investigation of the underlying mechanism between learning adaptability and SRL is of great significance to help to understand how to support students in successfully coping with the new challenges brought by educational reform and promote effective adjustment to challenges, including the “double reduction.”

To address the aforementioned issues, this study use a cross-sectional design to examine relations between learning adaptability and SRL under the “double reduction” background, examining academic motivation and self-management as mediators in a sample of Chinese junior high school students. As a first endeavor to clarify the mechanism underlying the relationship between learning adaptability and SRL, this investigation will deepen our understanding of how learning adaptability is linked to SRL and will inform intervention programs in teaching practice to enhance junior high school students’ SRL. Additionally, this study will also benefit future academic development research and educational intervention for different student groups by providing new insights and implications.

## 2. Learning adaptability and self-regulated learning

The shift to the new learning model created many academic pressures and challenges those students had to cope with. It is forecasted that the skills and competencies necessary for learning success in the accelerated changing academic context need to be founded upon adaptability, coping, and self-regulated learning (Otaki et al., 2022). From the perspective of adaptability, changes, uncertainty, and novelty can be “positive things and opportunities for growth and new beginnings” (Martin et al., 2017). Students with high learning adaptability have strong flexibility according to changes in the learning environment and can actively regulate their physical and existing cognitive schemata and change their learning behavior to achieve balance.

To adapt to the new learning requirements, as a driver of psychological development, learning adaptability allowed students to adapt to unfamiliar situations (the “double reduction” learning environment) efficiently and to activate self-regulated strategies, maintaining control of uncertainty, reducing the impact of stressors. Studies on the direct link between learning adaptability and SRL already exist but are sporadic, other analogous studies found evidence of the positive role of adaptability on SRL (Feraco et al., 2021, 2022; Guo, 2022; Otaki et al., 2022), from this perspective, learning adaptability and SRL become intertwined. One study on improving learners’ autonomous learning ability pointed out that a multilateral interactive teaching model can improve college students’ autonomous learning ability (in terms of SRL) through good learning adaptability (Xu et al., 2021). A recent study further found that learning adaptability is the essential personal resource that explains students’ confidence in activating SRL, in other words, students who demonstrated high levels of learning adaptability assigned and followed their own learning objectives and also set task strategies, environment structuring, time management (Stan et al., 2022). Hence, it has been established that learning adaptability plays an important role in helping students to facilitate their process of self-regulated learning, enhancing their confidence in coping with academic challenges.

## 3. Underling mechanisms between learning adaptability and self-regulated learning

Referring to academic learning, researchers have stressed the agentic role of learners, which lies in an intraindividual system. This internal system proposed by Ben-Eliyahu (2019) includes all the internal factors that drive the “individual’s trajectory through development” (Ben-Eliyahu, 2019). These internal factors include not only school-specific features such as academic motivation, self-management and SRL but also more general features like individual learning adaptability. The self-regulatory nature of learning adaptability indicates that it might favor specific study-related self-regulatory processes, such as SRL strategies, academic motivation, and self-management in the school learning setting. The intraindividual system provides a firm theoretical framework that combines learning adaptability with other individual features involved in academic learning. Therefore, in this study, we propose that academic motivation and self-management independently and sequentially mediate the relationship between learning adaptability and SRL.

### 3.1. Academic motivation as a mediator

Academic motivation is again a broad construct indicating an individual’s internal drive to achieve a certain goal, and many models of academic motivation have been proposed (Pintrich, 2003; Kriegbaum et al., 2018). Feraco et al. (2022) considered self-efficacy, theories of intelligence, and learning goals as the main components of academic motivation. These three concepts are related to SRL (Richardson et al., 2012; Burnette et al., 2013), and they can be considered a single factor representing students’ motivation (Mega et al., 2014). Several studies have reported academic motivation as a factor that can predict learners’ SRL skills (Kizilcec et al., 2017) and is influential in cultivating a self-regulated learner (Kalman et al., 2020). Xu et al. (2021) noted that autonomous learning (in terms of SRL) is a learner’s “ability to be responsible for their own learning.” This ability is influenced by many factors, including the learner’s own internal factors, such as learning attitudes and motivations (Xu et al., 2021).

More recently, models of SRL have emphasized on the impact of motivational factors, which have been seen as a direct part of SRL (Ben-Eliyahu, 2019) or as a prerequisite for successful SRL (Panadero, 2017). One study revealed that students who feel more positive achievement emotions also generally have higher academic motivation and a better use of SRL strategies, and more motivated students adopt better SRL strategies—and vice versa (Feraco et al., 2022). In line with this reasoning, learners will not achieve successful SRL with insufficient motivation for the task at hand (Jansen et al., 2019). We adhere to the latter definition, which excludes academic motivation to learn from the scope of the current review.

Researchers have studied the relationship between learning adaptability and academic motivation from various perspectives. Several studies have confirmed that students with intrinsic academic motivation establish goals such as learning and achievement goals and find it easier to adapt to educational environments (Cho et al., 2021; Raza et al., 2021). Moreover, research shows that adaptability can predict academic outcomes, such as motivation and engagement (Martin et al., 2013), and path analysis has indicated that adaptability positively and significantly predicts motivation (Martin et al., 2017; Feraco et al., 2021). Additionally, SRL is also an indicator of the fit between individuals and their learning environments, and the goodness of fit is addressed as a learning adaptation result. Therefore, this study assumed that academic motivation could be encouraged by learning adaptability and can mediate the association between learning adaptability and SRL.

### 3.2. Self-management as a mediator

According to Garrison’s self-directed learning (SDL) model, self-management is a contextual process that focuses on the task control issues of the external environment and activities, including managing the learning resources, support, and enactment of learning goals (Garrison, 2016). Its importance has also been recognized by the OECD and EU, and self-management was listed among the important core competencies of Chinese students (Lin, 2017).

Researchers widely agree upon the pivotal role of adaptability and self-management in students’ career development and regard them as important skills needed in the 21st century (Wrahatnolo and Munoto, 2018). However, the link between learning adaptability and self-management has remained virtually unexplored, but there is some evidence to suggest that for learners to be sufficiently skilled to function at a level at which they are self-directed, teachers need to promote students’ resilience and adaptability (Blignaut and Du Toit-Brits, 2022). Therefore, we ratiocinate that learning adaptability is correlated with self-management to a certain extent. Students who have a good ability to adapt to new learning requirements and curriculum plans would have a good sense of learning task control that is necessary for self-regulated learning.

Some researchers have recently examined how self-management denoting agentic capacity affects learners’ successful learning. Alario-Hoyos et al. (2017) found that learners need self-management to improve their self-regulation. Moreover, Onah et al. (2021) also explored learners’ self-directed learning abilities in MOOCs, and the results underscored learners’ self-management and its effects on self-regulation skills. In summary, we propose that junior high school students’ learning adaptability is associated with self-management and that self-management is further related to self-regulated learning, suggesting that self-management may serve as a mediator in the learning adaptability-self-regulated learning relationship.

### 3.3. Academic motivation and self-management

Not only are academic motivation and self-management related to self-regulated learning, but studies have also found that academic motivation and self-management are interrelated (Onah et al., 2021; Zhu and Doo, 2022). This suggests that academic motivation and self-management may interact and then promote self-regulated learning. In this study, two potential mediating effects should be considered. Self-management promotes self-regulated learning through academic motivation, and academic motivation affects self-management, which in turn increases self-regulated learning. The latter seems reasonable, as it was found that learners’ academic motivation mediated the relationship between self-monitoring and self-management in a learning setting (Abd-El-Fattah, 2010). This finding shows that it is more feasible to consider academic motivation as a factor for self-management than the opposite. This view is further supported by Zhu and Doo (2022), who investigated the association among motivation, self-monitoring, self-management, and MOOC learners’ use of learning strategies. The results showed indirect effects of motivation on self-management through self-monitoring. Based on previous research, we suppose that learning adaptability contributes to SRL via the sequentially mediated effects of academic motivation and self-management for junior high school students.

## 4. Hypotheses

**Figure 1:**
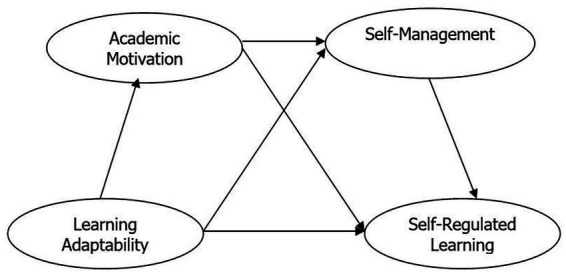
Proposed research model.

The hypothetical structure of the relations between the variables, taking SRL as the main dependent variable and the others as predictive variables, was based on the predictors’ “specificity” to SRL (see Figure 1). Therefore, we propose the following hypothesis:

*Hypothesis 1:* Students’ learning adaptability and SRL, which SRL can be positively predicted by learning adaptability.

*Hypothesis 2:* Academic Motivation mediates the relationship between learning adaptability and self-regulated learning for junior high school students.

*Hypothesis 3:* Self-management will play a mediating role in the relationship between learning adaptability and self-regulated learning for junior high school students.

*Hypothesis 4:* The academic motivation and self-management will play a chain mediating role in the relationship between learning adaptability and self-regulated learning for junior high school students.

## 5. Materials and methods

### 5.1. Participants

Research data were collected via Wenjuanxing (WJX),^1^ based on a convenience sampling method, 893 junior high school students voluntarily participated in the assessment in two provinces (cities): Guangzhou and Zigong. After excluding invalid data with incorrectly reported information (such as a filling time that substantially deviated from the average filling time, choosing the same option, and providing zigzag answers), we noted 787 valid questionnaires. Table 1 shows the participants’ demographic information.

**Table 1:** Participants’ descriptive statistics by school year.

School year	7	8	9	Total
Number	333	245	209	787
Females	160	135	122	417
Males	173	110	87	370

### 5.2. Measures

#### 5.2.1. Learning adaptability

We adapted the subscales of the Learning Adaptation Scale (Gao, 2019) to assess students’ adaptability. This scale contains seven items for motivation (e.g., “I find studying boring”), seven items for learning skills (e.g., “I have the habit of actively previewing or reviewing my lessons”), four items referring to learning efficacy (e.g., “I believe I have the ability to obtain good academic achievement”) and four items for learning problems (e.g., “I tend to lose concentration when studying”). The items were rated on a 5-point scale ranging from 1 (never) to 5 (always). Each scale (except for the learning skills and learning efficacy scales) presented negative items, and the total score was calculated as the sum of the ratings for each item (after reversing the score for the negative items). In the present study, two items were dropped due to factor loadings below 0.6, composite reliability (CR) was 0.96, and confirmatory factor analysis (CFA) also showed that the measurement model yielded an adequate fit (χ^2^/df = 2.66, CFI = 0.96, TLI = 0.95, RMSEA = 0.05, SRMR = 0.04), indicating good construct validity.

#### 5.2.2. Self-management

We used the Self-Management Scale (Meng et al., 2011) to assess the students’ self-management in terms of social management (e.g., “You are usually one of the main organizers of class gatherings”), knowledge time management (e.g., “You will take the initiative to ask teachers or classmates for questions you are confused about”) and mental health management (e.g., “Are you satisfied with your academic achievement”). The Self-Management Scale was adequately reliable in the original study (*α* = 0.89). In the current study, the scale on a 5-point Likert scale from 1 (very inconsistent) to 5 (very consistent), showed high reliability, CR was 0.91, and CFA also showed that the measurement model yielded an adequate fit (χ^2^/df = 3.96, CFI =0.94, TLI = 0.93, RMSEA = 0.06, SRMR = 0.03), indicating good construct validity.

#### 5.2.3. Self-regulated learning

SRL was captured using the Self-Regulated Learning Strategy Questionnaire (Kong and Lu, 2012). It consists of seven subscales: self-efficacy (seven items; e.g., “I am sure that I can successfully apply what I have learned to solve problems”), meta-cognitive strategies (six items; e.g., “I try to change my reading methods when I encounter difficult reading materials”), cognitive strategies (five items; e.g., “I use drawings, lists, etc., to sort what I have learned”), extrinsic motivation (four items; e.g., “I want to get good grades on the exam, because it is very important for me to show my ability in front of my family and classmates”), academic emotion regulation strategies (four items; e.g., “I try to cheer myself up when I am depressed because of my unsatisfactory exam results”), intrinsic motivation (two items; e.g., “I am interested in the specific content of a certain subject”), and cooperative learning strategies (three items; e.g., “I often discuss problems in my study with my classmates”). The participants rated the frequency of each item on a scale ranging from 1 (very inconsistent) to 5 (very consistent), and with higher scores indicating greater SRL ability. In the present study, CR was 0.95, and CFA also showed that the measurement model yielded an adequate fit (χ^2^/df = 4.32, CFI = 0.92, TLI = 0.92, RMSEA = 0.07, SRMR = 0.05), indicating good construct validity.

#### 5.2.4. Academic motivation

The participants completed the Academic Motivation Scale (Dong, 2021). This self-report questionnaire measures students’ academic motivation (e.g., “I feel welcome at this school”). The participants rated the accuracy of each item on a 3-point Likert scale ranging from 1 (never want) to 3 (always want). In the present study, the scale showed high reliability, CR was 0.81, and CFA also showed that the measurement model yielded an adequate fit (χ^2^/df = 3.75, CFI = 0.98, TLI = 0.97, RMSEA = 0.06, SRMR = 0.03), indicating good construct validity.

### 5.3. Procedure

A cross-sectional design was used. Data for this study were collected via the online questionnaire service platform of WJX, which has been widely used for Chinese academic institutions and individuals due to its obvious advantages of reliable, easy to use and efficient. On the premise of informed consent, participants were required to complete all questions of the online questionnaire. All questions were compulsory and had fixed scores, by which missing data and outliers were avoided. All participants were well briefed that their participation was voluntary, that withdrawal could be allowed at any time, and the information collected would be confidential and used only for scientific research.

### 5.4. Data analysis

IBM SPSS 23 was used to conduct the preliminary analyses. We applied structural equation modeling (SEM) in Mplus7.0 (Muthén and Muthén, 1998–2017) to assess the relations between variables with maximum likelihood estimation. The following five fit indices were chosen for analyses: CHI-SQR/DF, comparative fit index (CFI), Tucker–Lewis index (TLI), root mean square error of approximation (RMSEA), and standardized root mean residual (SRMR). In this study, considering our sample size, model complexity, and the reliability of the measures, we adopted Hu and Bentler’s (1999) less conservative recommended fit values: CFI > 0.90; TLI > 0.90; RMSEA <0.10; and SRMR <0.08; 1 < CHI-SQR/DF < 3.

## 6. Results

### 6.1. Data screening and preliminary analysis

As all questions were compulsory and had fixed scores; there were no missing data or outliers, so we directly examined the distributions. The SPSS outputs showed that the absolute values of skewness and kurtosis of all study variables ranged from −2 to 2, suggesting a normal distribution (George and Mallery, 2010). Correlations were tested to determine the relations among learning adaptability, SRL, self-management, and motivation. Table 2 shows the results. In line with our hypotheses, all variables were positively correlated. All these correlations show that it is doable to further analyze mediation effects (Frazier et al., 2004).

**Table 2:** Correlations, convergent validity, and discriminant validity.

DIM.	Convergent validity			Discriminant validity			
	Std. loading	CR	AVE	LA	SRL	SM	AM
LA	0.67–0.85	0.96	0.55	**0.74**			
SRL	0.69–0.92	0.95	0.73	0.67^**^	**0.86**		
SM	0.85–0.90	0.91	0.77	0.58^**^	0.79^**^	**0.88**	
AM	0.63–0.76	0.81	0.45	0.42^**^	0.56^**^	0.56^**^	**0.67**

Figures in bold are the square root of AVE, and Pearson correlation is in the lower triangle. *N* = 787. LA, learning adaptability; SRL, self-regulated learning; SM, self-management; AM, academic motivation. **p < 0.01.

### 6.2. Measurement model

Before conducting the structural model, four CFAs were performed using maximum likelihood estimation to test convergent validity and discriminant validity (see Table 2). Convergent validity is checked in the following three ways (Hair et al., 2009). First, the CFA results for all standardized factor loadings were over 0.6, and the measurement model indicated a good fit for the data. Second, the average variance extracted (AVE) values were over 0.5. Third, all CR values of the constructs were over 0.7. The results confirmed that the measurement model has good convergent validity. Discriminant validity was examined using the square root of AVE for comparison with the correlation coefficient of each latent variable. As shown in Table 2, the values of the square root of the AVE were larger than each latent variable’s correlation coefficient, indicating acceptable discriminant validity.

Considering that the three variables of learning adaptability, SRL and self-management in this study are multidimensional and multi-indicator constructs, on the premise that the first-order model fits well, for the purpose of model precision, this study replaces the first-order model with a second-order model for the next SEM analysis by calculating the target coefficient (T), which is the ratio of the chi-square of the first-order model to the chi-square of the second-order model. The target coefficient has an upper limit of 1, which is possible only if the relations among the first-order factors can be totally accounted for in terms of the more restrictive model (Marsh and Hocevar, 1985). The T values of SRL, learning adaptability, and self-management are 0.84, 0.85, and 0.99, respectively, which are close to 1 in this study. The fitness index of the second-order CFA of SRL, learning adaptability, and self-management revealed good fitness. Therefore, this study takes the results of second-order CFA to implement structural model analyses.

### 6.3. Structural model

**Figure 2:**
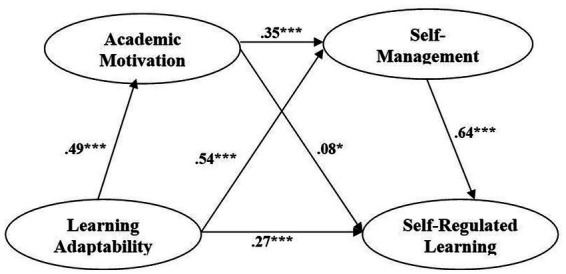
Standardized parameter estimates of the effects of learning adaptability on SRL mediated by academic motivation and self-management.

We tested the hypotheses including all direct and indirect paths with the constructed structural model. The results indicated structural model fit statistics of χ^2^/df = 2.67; TLI = 0.9; CFI = 0.9; SRMR = 0.06; and RMSEA = 0.05 (Browne and Cudeck, 1992; MacCallum et al., 1996). Figure 2 and Table 3 show that all the model paths were significant. Learning adaptability and SRL were significantly correlated (*p* < 0.001); moreover, the former significantly predicted the latter (*β* = 0.28, *p* < 0.001). The direct paths from learning adaptability to motivation (*β* = 0.29, *p* < 0.001) and self-management (*β* = 0.56, *p* < 0.001) were both significant. Both motivation (*β* = 0.15, *p* < 0.05) and self-management (*β* = 0.64, *p* < 0.001) were significantly positively related to SRL.

**Table 3:** Research model regression weight.

DV	IV	Estimate	S.E.	Z	*P*-value	*R*^2^
SRL	SM	0.64	0.06	10.60	***	0.82
	AM	0.15	0.07	2.03	*	
	LA	0.28	0.05	5.27	***	0.60
SM	LA	0.56	0.07	8.36	***	
	AM	0.62	0.10	6.14	***	
AM	LA	0.29	0.03	9.15	***	0.24

LA, learning adaptability; SRL, self-regulated learning; SM, self-management; AM, academic motivation. *p < 0.05; ***p < 0.001.

In the current study, 1,000 bootstrapping samples and bias-corrected confidence intervals were adopted to test indirect effects in the model (Hayes, 2009). There were four significant indirect relationships (Table 4). Learning adaptability had a significant indirect impact on SRL via motivation and self-management. Learning adaptability had a significant indirect impact on self-management via motivation. Moreover, motivation and self-management played a chain mediating role in the relationship between learning adaptability and self-regulated learning.

**Table 4:** Totals, total indirect, specific indirect and direct effects.

Path	Estimate	S.E.	Est./S.E.	*P*-value	95% C.I.
Effects from LA to SRL					
Total	0.79	0.06	13.50	***	[0.66 0.90]
Total indirect	0.52	0.05	10.67	***	[0.43 0.62]
Specific indirect					
LA → AM→SRL	0.04	0.02	1.96	*	[0.00 0.09]
LA → SM → SRL	0.36	0.05	7.64	***	[0.28 0.47]
LA → AM→ SM → SRL	0.11	0.02	4.97	***	[0.08 0.16]
Direct					
LA → SRL	0.28	0.05	5.27	***	[0.18 0.38]
Effects from LA to SM					
Total	0.74	0.06	11.92	***	[0.62 0.86]
Total indirect	0.18	0.03	5.91	***	[0.13 0.24]
Specific indirect					
LA → AM →SM	0.18	0.03	5.91	***	[0.13 0.24]
Direct					
LA → SM	0.56	0.07	8.36	***	[0.44 0.70]

LA, learning adaptability; SRL, self-regulated learning; SM, self-management; AM, academic motivation. *p < 0.05; ***p < 0.001.

## 7. Discussion

This study tested how learning adaptability relates to SRL and how this ability works together with academic motivation and self-management as part of a single intraindividual system. The findings support the four hypothesized direct and indirect effects: (1) learning adaptability→SRL, (2) learning adaptability→academic motivation→SRL, (3) learning adaptability→self-management→SRL, and (4) learning adaptability→academic motivation→self-management→SRL.

This study revealed that learning adaptability represented the essential personal psychological resource, a facilitator of junior high school students’ SRL in different learning models, even in new situations implying uncertainty. In our sample, the “double reduction” learning model provides students with the opportunity to independently control when, what, and how to learn. The rapid and efficient learning adaptation makes students show higher self-efficacy and full of confidence in their learning ability, take initiative in self-regulated learning. In this process, students will independently set and formulate learning goals and learning plans, adopt appropriate learning strategies, supervise the whole learning process, and constantly compare and correct the gap between the current state and the target state; meanwhile, they will actively adjust and integrate external resources to achieve the predetermined learning goals successfully. It can be seen that students with high level of learning adaptability have stronger self-confidence, so they are more likely to set higher tasks and requirements in learning, face up to the difficulties encountered in learning and solve them positively in the process of learning, showing stronger monitoring and self-regulation learning ability. This result was generally in line with our hypothesis and previous research about the link between learning adaptability and SRL (Xu et al., 2021; Stan et al., 2022) and provides new empirical evidence for the notion that this link lasting as the learning model changes.

Academic motivation was a significant mediator for learning adaptability to SRL, as the result revealed. This finding supports the assumption that learning adaptability describes general psychological feature that adjust cognition, behaviors and affections contributing to students’ academic motivation at school, and favoring SRL. Thus, consistent with Jansen et al. (2019), it seems that students’ psycho-behavioral adjustments in learning process contribute to the perceptions of performance capabilities, task value and goal orientation that form the basis of motivational beliefs. Analyzing the contribution of academic motivation to SRL indicated that students reported higher levels of efficacy, interest and value about the learning task more cognitive strategies use, like rehearsal, elaboration, and organizational strategy to monitor and regulate their cognition. This work on academic motivation fits nicely with self-regulated learning theory because it is assumed that in order for students to self-regulate their learning, performance, and behavior, they must have some task value beliefs, perceived competence, and goal orientations against which to evaluate the importance of the task, judge their ability to accomplish learning task, and provide goal or standard or criterion for comparing their progress. In this sense, academic motivation is a core factor in the path of students’ academic development, and incorporating the role of academic motivation into the linkage between learning adaptability and SRL may help shed new light on how this relationship works.

Compared with academic motivation, self-management was found to play a more important role in explaining the association between learning adaptability and SRL, serving as a significant mediator for learning adaptability to SRL. Learning adaptability as a student’s psychological resources that represents one’s self-regulatory ability toward learning tasks will induce various beliefs or behavioral responses on how to cope with learning tasks. Operational indicators of learning adapting responses include self-management or learning planning. Students who had a good adaptability resource to adapt to new learning requirements and curriculum plans had high degrees of initiative in using self-managing behaviors, in which process students were encouraged to adopt self-regulated learning strategies to direct, control, and regulate the process of collecting information, planning to solve problems and making decisions. The results are in line with our hypothesis about the link between learning adaptability and self-management and are consistent with the conclusion that self-management is significantly associated with SRL (Alario-Hoyos et al., 2017; Onah et al., 2021). Self-management is an increasingly valuable agentic capacity in the ever-changing learning world, and its mediating role in the relationship between learning adaptability and SRL provides new insights into how this relationship occurs and further highlights the importance of agentic capacity strength in the SRL process.

Finally, it is worth mentioning that learning adaptability is indirectly linked with SRL through the chained mediation path of academic motivation and self-management. As in previous studies, academic motivation may shape self-management, and there were predictive effects between the two (Zhu and Doo, 2022). Theoretically, as a well-adjusted student would possess high coping ability in the field of study and develop a high sense of self-efficacy, he or she would feel more capable and have better planning, self-management, and self-regulation in academic study. This study revealed that students who reported an adaptability to comply with the novel and challenging learning model manifested high levels of willingness to invest sustained effort into academic task management, which in turn facilitated SRL to concentrate their cognitive resources at hand on the current academic tasks, keep track of their academic work, adjust their learning strategies through reflection and comparison, and ultimately achieve the predetermined learning goals smoothly. These results show that not only are academic motivation and self-management the cornerstones of SRL, but the sequential effect also explain why learning adaptability is related to SRL. These findings deepen the correlation between academic motivation and self-management by exploring more possible paths to understand the connection between learning adaptability and self-regulated learning and expand the previous theoretical proposition by providing more comprehensive insight into how these two psychological characteristics can be integrated to promote students’ academic development.

In sum, this study is consistent with the previous studies regarding learning adaptability as the malleable personal characteristics of the intraindividual system, which promotes SRL together with other components of the intraindividual system. The key contribution of this study is to provide new insights into the literature that academic motivation and self-management separately and sequentially mediate the learning adaptability, SRL links, and learning adaptability is effective driver of SRL in the population of junior high school students.

## 8. Limitations

The findings presented above should be seen in light of several limitations that will inspire future research. First, since learning adaptability is important for individuals’ learning outcomes, subsequent educational choices and opportunities, and their future development, longitudinal analysis should be conducted to link their success in school education with their career achievements to study the various influences of learning adaptability throughout a person’s course of studies and work career. Second, the relationship between learning adaptability and SRL remained significant after the introduction of motivation and self-management as mediators, which only partially explained the effect, future research may focus on other variables, such as contextual variables, prompting the further understanding of the association between learning adaptability and SRL by connecting the contextual factors with in-person factors. Third, the data of this study were self-reported via an online questionnaire, and Harman’s single-factor test confirmed that there was no common method variance in our data (i.e., total variance for a single factor was 34.31). However, to strengthen and verify the research results, more data collection methods are strongly recommended for future research, such as interviews and report data from others.

## 9. Conclusion

The main aim of the present study is to understand how learning adaptability is related to SRL among junior high school students. This study has explored the association between learning adaptability, academic motivation, self-management, and SRL and found that learning adaptability relates to SRL, and further, academic motivation and self-management partially and chained mediated the association between learning adaptability and SRL. We hope these results promote future theoretical research and educational intervention on SRL of junior high school students and help them smoothly adapt to the new teaching model brought about by the “double reduction” and complete the transition to higher education.

## Data availability statement

The raw data supporting the conclusions of this article will be made available by the authors, without undue reservation.

## Ethics statement

Ethical review and approval was not required for the study on human participants in accordance with the local legislation and institutional requirements. Written informed consent to participate in this study was provided by the participants’ legal guardian/next of kin.

## Author contributions

CS and QX: conceptualization. QX, QL, and CS: methodology. CS: software, formal analysis, and writing—original draft preparation. CS, QX, and WJ: validation, investigation, and resources. CS and WJ: data curation. CS, QX, QL, and WJ: writing—review and editing. All authors contributed to the article and approved the submitted version.

## Funding

This study was supported by the 13th Five-Year Plan Project of National Education Science (BBA200033) and Neuroeconomics Laboratory of Guangzhou Huashang College (2021WSYS002).

## Conflict of interest

The authors declare that the research was conducted in the absence of any commercial or financial relationships that could be construed as a potential conflict of interest.

## Publisher’s note

All claims expressed in this article are solely those of the authors and do not necessarily represent those of their affiliated organizations, or those of the publisher, the editors and the reviewers. Any product that may be evaluated in this article, or claim that may be made by its manufacturer, is not guaranteed or endorsed by the publisher.

## Footnotes

^1^https://www.wjx.cn/
